# Reduced dynamical maps in the presence of initial correlations

**DOI:** 10.1038/srep37328

**Published:** 2016-11-22

**Authors:** Bassano Vacchini, Giulio Amato

**Affiliations:** 1 Dipartimento di Fisica, Università degli Studi di Milano, Via Celoria 16, Milan, 20133, Italy; 2 INFN, Sezione di Milano, Via Celoria 16, Milan, 20133, Italy

## Abstract

We introduce a framework for the construction of completely positive dynamical evolutions in the presence of system-environment initial correlations. The construction relies upon commutativity of the compatibility domain obtained by considering the marginals with respect to the environmental degrees of freedom of the considered class of correlated states, as well as basic properties of completely positive maps. Our approach allows to consider states that can have finite discord, though it does not include entangled states, and it explicitly shows the non-uniqueness of the completely positive extensions of the obtained dynamical map outside the compatibility domain. The possible relevance of such maps for the treatment of open quantum system dynamics is critically discussed, together with the connection to previous literature.

A ubiquitous situation in quantum physics involves the description of systems which are not isolated, so that their dynamics is actually influenced by other quantum degrees of freedom. The theory of open quantum systems was indeed developed to cope with such situations and has found important applications in diverse fields starting from quantum optics to condensed matter theory, chemical physics and many others^1^,^2^,^3^. The standard description of an open quantum system dynamics rests on two basic assumptions, namely an initial system-environment state in factorized form and weak coupling between system and environment. In such a case the existence of a reduced dynamics is granted and it can reasonably be taken to obey a semigroup composition law in time, so that the most famous result by Gorini, Kossakowski, Sudarshan and Lindblad applies, fully characterizing the generator of such a dynamical semigroup^4^,^5^. A lot of effort has been devoted to overcome these limitations within the standard framework in which the reduced open quantum system dynamics is obtained by tracing over the environmental degrees of freedom, but also different approaches have been recently proposed^6^.

Major results have been obtained in describing dynamics beyond the weak coupling limit, leading to reduced dynamical evolutions which go beyond a simple semigroup composition law. These strong coupling dynamics often show up memory effects. Indeed also in this respect important results have been obtained in providing a characterization of non-Markovian dynamics within open quantum system theory, and all of these results rely on the existence of a reduced system dynamics^7^,^8^. On the contrary, despite important efforts^9^,^10^,^11^,^12^,^13^,^14^,^15^,^16^,^17^,^18^,^19^, the extension of the formalism to go beyond initially factorized states appears to be much harder, and a general satisfactory treatment still lags behind. Indeed such an extension is called for, since the choice of factorized initial states is well compatible with a weak coupling approach, but is generally not a natural assumption if one considers situations in which the coupling between system and environment degrees of freedom is actually strong. The question of whether and insofar one can consider a reduced system dynamics in the presence of initial correlations is of major importance, as also shown by the great amount of literature devoted to the subject. Indeed if such an approach is not feasible, or only restricts to quite special cases as shown in this article, one has to look for new strategies in order to extend an open quantum system description to such situations.

Different paths have been followed in order to tackle the issue of initially correlated states between system and environment in special systems of physical interest^20^,^21^,^22^,^23^,^24^. In particular great attention has been devoted to study the conditions under which a completely positive map can in some sense be introduced. The difficulties and possible inconsistencies arising in trying to consider a reduced dynamical description were first considered in refs 25 and 26. In these seminal papers the trouble in combining the basic key issues of linearity, complete positivity and consistency of the description in the presence of correlations already appeared. To solve this issue^25^ suggested relaxing positivity, while^26^ pointed to the possibility of restricting the domain on which the assignment map linking the reduced system state to the total state is physically meaningful. The issue of relating the definition of a reduced dynamical evolution to a suitable domain of reduced system states thus first entered the debate. An analysis of the dependence of the evolved state on the initial correlations was performed by means of a definite example in ref. 27, where it was stressed that the characterization of the correlations was indeed necessary in order to correctly predict the reduced system time evolution, also pointing to how such correlations might be included in the equation of motions. The situation in which the interaction Hamiltonian is kept fixed has been explored in ref. 16, looking for linearity and positivity of the maps connecting the reduced initial state with the time evolved one, in their dependence on the initial correlations. However it is of course of interest to study the existence of a dynamical map for correlated states allowing for a generic interaction between system and environment. In ref. 9 the focus thus went back to the evolution of correlated states evolving through a general Hamiltonian. In the paper the authors pointed to a Kraus construction of the time evolution map for zero discord system-environment states, thus warranting complete positivity. In a subsequent paper^10^ it was claimed that a completely positive reduced dynamics could be introduced if and only if one considered zero discord states. The claim was however later reconsidered and retracted by the author themselves in refs 17 and 18, where they also explored the introduction of different inequivalent notions of complete positivity in order to provide a possibly larger framework, still stressing that the problem remained open. In particular this work did not point out a particular class of possible correlations allowing for a reduced description, except for zero discord states. States with non zero quantum discord were first considered in ref. 12 providing a specific counterexample to ref. 10. The counterexample was based on a Kraus representation for the transformation in time of the state providing the reduced system dynamics. In ref. 14, at variance with previous work mainly focussed on the Kraus decompostion, the important issue of linearity as a premise for complete positivity was clearly stated, though the authors were mainly concerned with discussing the role of quantum discord in the characterization of complete positivity. Other approaches have been put forward^13^,^15^,^19^, however allowing for the introduction of ancillary systems or for different system-environment partitions of the Hilbert space at initial and final time. While the variety of devised approaches does not always allow for a straightforward comparison, the abovementioned results share the idea of looking for a proper definition of a map describing the reduced system dynamics.

Indeed in the usual framework of open quantum systems^1^ one considers a tensor product structure 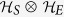, where 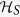 and 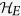 denote the Hilbert space of system and environment respectively, and assumes that the overall system is closed, so that its time evolution can be described by a group of unitary operators *U*_*SE*_(*t*). Within this description, given an arbitrary initial system-environment state *ρ*_*SE*_(0), with corresponding reduced system state *ρ*_*S*_(0) = *Tr*_*E*_*ρ*_*SE*_(0), where *Tr*_*E*_ denotes the partial trace with respect to the environment degrees of freedom, one can naturally consider the following collection of time dependent transformations


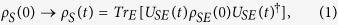


which by construction preserve positivity and trace. If the initial state *ρ*_*SE*_(0) is actually factorized, so that *ρ*_*SE*_(0) = *ρ*_*S*_(0) ⊗ *ρ*_*E*_(0), it is well known that in such a way one obtains a linear map defined on the whole set of states, which in particular can be shown to be not only positive, but actually completely positive. The notion of complete positivity^28^,^29^ naturally emerges in this open quantum system setting and is indeed a typical quantum feature, related to the tensor product structure of the space describing a composite system. At the level of states one can witness the difference between positivity and complete positivity of a map by the application to entangled states, while at the level of observables the same difference can be appreciated by applying the map to non commuting set of observables. Indeed it is an important result that for a map acting on a commutative space positivity is equivalent to complete positivity^30^,^31^. More precisely the notions of positivity and complete positivity coincide if either starting or arrival space of the map is given by a commutative algebra, which therefore is amenable to a classical description^32^.

In this article we will build on these basic facts to point out a construction of completely positive maps arising in the presence of a correlated system environment state. As we shall see this approach allows us to recover as special cases some results previously obtained in the literature^9^,^12^ and clarifies the general framework to which they belong. It further shows the severe limitations encountered when extending the formalism of open quantum systems to the situation of initially correlated states. It appears as a first key fact that the relevant sets of system states, namely separable states with commuting marginals on the side of the system, actually admit a classical characterization. In particular as a second key fact we will show that, even when a completely positive map can be defined in the presence of initial correlations, its application besides a domain of commuting system states, which in this sense can be described as classical domain, is far from unique and upon extension no more allows for a clear dynamical interpretation. These results appearing for the first time in the present approach thus clarify the common root of special results previously obtained in the literature and call for new approaches, as also advocated in refs 22 and 33.

## Results

### Construction of quantum maps starting from correlated states

In order to consider the possibility to introduce completely positive maps starting from correlated system environment states let us first consider the following class of correlated states


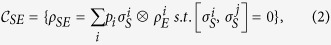


where {*p*_*i*_}_*i*_ is a probability distribution, the 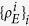 a collection of states for the environment, 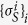 a fixed set of commuting statistical operators for the system and we assume the Hilbert space of the system 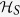 to be finite dimensional with dimension *n*. The set 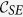, containing separable states with commuting system marginals, provides a convex subset of the whole set of states on 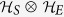, which we denote by 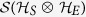. In particular it is a subset of the set of separable states which includes also zero discord states^34^,^35^. To this set we can associate a compatibility domain given by the set of system states which can be obtained as marginals of these correlated states, namely


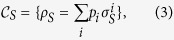


which is still a convex set and is in particular a commutative set. Note however that the relationship between sets of correlated states and their compatibility domain is many to one, so that the very same compatibility domain may arise from different classes of separable correlated states. The set 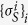 is generated by a set of 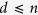 statistical operators with orthogonal support, where *d* is the dimension of the linear hull of 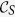. If *d* = *n* in particular 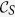 is given by the convex combinations of states which are all extremal. Supposing *d* < *n*, so that at least one of the 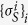, say *W*, is not necessarily a projection operator, without loss of generality we have


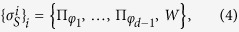


where {*φ*_*i*_}_*i*=1,…,*d*−1_ are orthonormal vectors in 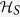 and we have introduced the one dimensional projections 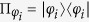. The statistical operator *W*, which is not a pure state, admits many different decompositions. In particular one can consider an orthogonal decomposition


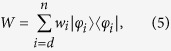


given by its spectral resolution, where the {*φ*_*i*_}_*i*=*d*,…*n*_ are orthonormal vectors, further orthogonal to the span of {*φ*_*i*_}_*i*=1,…,*d*−1_, so that altogether they provide a basis in 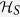, and the positive weights *w*_*i*_ sum up to one. At the same time one can consider many others non orthogonal convex decompositions of the form


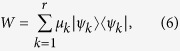


with *r* > *n* − (*d* − 1) and where the {*ψ*_*k*_}_*k*=1,…,*r*_ are normalized but generally non orthogonal states while {*μ*_*k*_}_*k*=1,…,*r*_ is a probability distribution. For a choice of system states of the form Eq. (4) the compatibility domain Eq. (3) can therefore be seen to arise from the two following distinct sets of correlated system environment states, namely


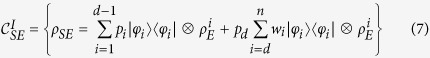


and


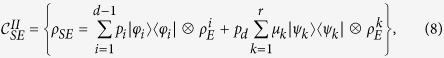


with 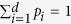, while 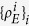 and 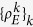 are collections of possibly distinct environmental states. While these composite states have the same compatibility domain according to Eq. (4), we have the important difference that while 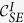 only contains zero quantum discord states, this is no more true for 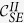, which thus also includes quantum correlations. Given an arbitrary system environment interaction *U*_*SE*_(*t*) we can now consider the transformation Φ^*II*^(*t*) which associates to the marginal of a state 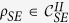, that is


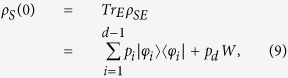


the marginal associated to the time evolved state according to


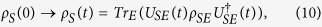


so that we set


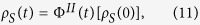


and an analogue construction can be done starting from states in 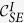, thus obtaining a collection of maps Φ^*I*^(*t*). We have now the important fact that such assignments actually define positive affine maps on the convex set 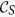, which can be uniquely extended to linear maps on the linear hull of 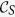. Since the elements of the set 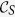 commute, according to refs 30 and 31 we therefore have that such maps are actually completely positive. We can now build on another fundamental result about linear maps which are completely positive, namely the fact that they can be expressed in the so-called Kraus form^36^.

To explicitly exhibit a Kraus representation for the considered maps we proceed as follows. Let us first evaluate the trace in Eq. (10) by considering a complete orthonormal system {|*ϒ*_*γ*_〉} in 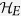, thus obtaining


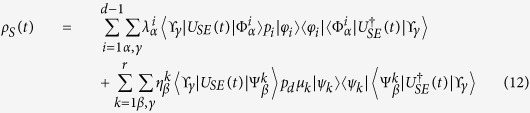


where we have also introduced orthogonal decompositions for the environmental operators appearing in Eq. (7) and Eq. (8) according to 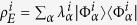 and 
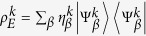
. We now want to recast Eq. (12) as a linear action on *ρ*_*S*_(0) as given by Eq. (9). To this aim we first observe that we have


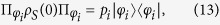


which allows us to express in the desired fashion the first line of Eq. (12). To proceed further we exploit a general theorem which connects different possible orthogonal and non orthogonal decompositions of a given quantum state. The theorem was first formulated by Schrödinger^37^ and later rediscovered by Gisin^38^ as well as Hughston, Josza and Wootters^39^, so that it is often known as GHJW theorem (see ref. 40 for a more detailed analysis of the history of the result). According to this theorem given the two decompositions Eq. (5) and Eq. (6) of the statistical operator *W* there exists a unitary matrix *U*, whose columns are given by 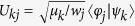 for *j* = *d*, …, *n*, so that in particular setting


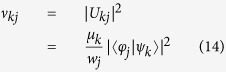


we have for all *k*


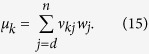


We can thus introduce the operators


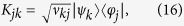


satisfying the relation


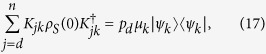


which allows to express the second line of Eq. (12) as a linear trasformation acting on *ρ*_*S*_(0). Thanks to Eq. (13) and Eq. (17) we can finally introduce the system operators


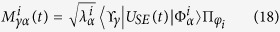


together with


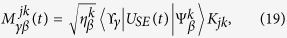


which provide an explicit Kraus representation of the map Φ^*II*^(*t*) defined through Eq. (12)


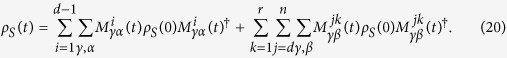


The obtained expression for the map Φ^*II*^(*t*) allows to extend it by linearity to the whole set of system states 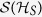 in Kraus form, thus remaining completely positive. This construction contains as special case the examples considered in refs 12 and 13.

Note that through this construction besides the collection of time dependent positive operator-valued measures naturally associated to the family of channels Φ^*II*^(*t*) thanks to trace preservation^28^, one can also put into evidence a positive operator-valued measure determined by the class of correlated system-environment states. The latter is given by the set 
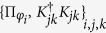
, where the indexes take on the values *i* = 1, …, *d* − 1, *j* = *d*, …, *n* and *k* = 1, …, *r*. It is actually fixed by the following transformation which leaves invariant the compatibility domain associated to 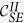



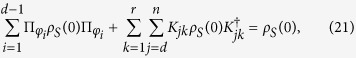


with *ρ*_*S*_(0) as in Eq. (9). The analogous construction for the family of channels Φ^*I*^(*t*), leads in particular to the projection-valued measure 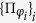, with *i* = 1, …, *n*.

We have thus provided a simple construction to define a completely positive map starting from a correlated system-environment state, which generally has non vanishing quantum discord, belonging to a convex subset of the whole set of states whose compatibility domain is actually a commutative set. In such a way for arbitrary system-environment interaction one obtains a positive map providing the dynamical evolution of the reduced system, which due to commutativity of the domain on which it is defined, namely the linear hull of the convex compatibility domain, has to be completely positive and therefore admits a Kraus representation. This construction therefore builds on the simple but telling identification of the properties of positivity and complete positivity on commutative domains, which shows the deep limitations in striving to obtain a completely positive dynamics for arbitrary system-environment interaction. In the essence apart from the case of factorized initial states one recovers a completely positive dynamics restricting to a classical ensemble of states, which commute among themselves. Given its expression in terms of Kraus operators the map can be extended as completely positive map to the whole set of reduced system states. As we shall stress below, this extension is in general highly non unique. This non-uniqueness of the extension, which therefore does not allow for a dynamical interpretation, naturally appears in our approach. It therefore puts into major evidence the basic inherent difficulties encountered in speaking of reduced dynamics in the presence of initial correlations. From a mathematical viewpoint this relies on the fact that the linear hull of the compatibility domain has dimension strictly less than the linear space containing the whole set of system states. Furthermore the very construction of the maps does depend on both the reduced system state, the environmental states and the considered correlations. In the case of a reduced system state with a degenerate spectrum in particular the same state can belong to compatibility domains arising from different correlated states, and system-environment states with the very same marginals lead to utterly different maps.

### Zero quantum discord states and non-uniqueness of the construction

A similar but simpler construction with respect to the one considered above can be obtained for zero quantum discord states, starting from the set 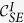, in analogy with the result obtained in ref. 9. In that paper it was first shown how to obtain the time evolution of a reduced system initially correlated with the environment, expressing the reduced system transormation as a map in Kraus form. We now extend this result putting into evidence the non-uniqueness of the completely positive extension of the maps, stressing the fact that they are initially defined only on the compatibility domain made up of commuting statistical operators for the system. This non-uniqueness of the extension outside the compatibility domain is indeed a basic limitation in looking for a reduced dynamics in the presence of initial correlations. This fact has not yet been put into evidence and clearly appears in our approach, in which one realizes that the very definition of the map leads to introduce a convex commutative domain, of measure zero within the whole set of states. Indeed considering a system statistical operator in the compatibility domain associated to 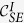, namely of the form


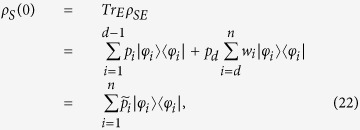


we can introduce two channels sending states in 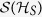 to states in 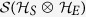, namely


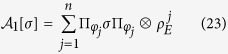


and


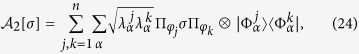


which coincide on states belonging to the compatibility domain. The two channels are written in Kraus form, as shown in the Methods Section, so that they can be extended from the linear hull of the compatibility domain to the whole linear space of trace class operators on 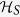, thus allowing to define the completely positive maps


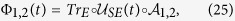


where 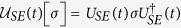. We note in particular that upon introducing the diagonalizing projection


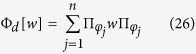


we have


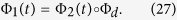


Considering the action of 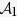 on states of the form Eq. (22) we obtain a representation of Φ_1_(*t*) in Kraus form as


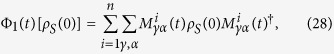


which is the analogue of Eq. (20) for states coming from 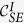. We can however also consider the channel map 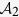 and thus come to the completely positive map


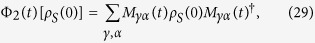


where we have defined another set of Kraus operators according to


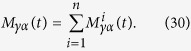


**Figure 1:**
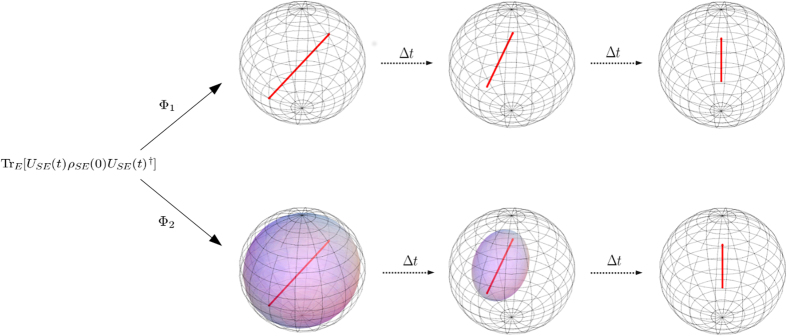
Schematic illustration of the different action of the maps Φ_1_(*t*) and Φ_2_(*t*) defined in Eq. (28) and Eq. (29) respectively. The maps are obtained for an initial correlated state of the form (35) with parameters 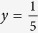 and 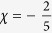. In the plots the external frame corresponds to the Bloch sphere, while the colored inset represents the image of the Bloch sphere upon the action of the maps at different times. The first plot in the sequence refers to the time *t* = 0, so that one sees that these maps only act as the identity at the initial time on the commutativity domain, given by the red diameter. This fact can be read directly from the expression Eq. (23) and Eq. (24) respectively of the assignment maps 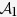 and 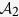, which lead to the definition of the maps Φ_1_(*t*) and Φ_2_(*t*). While the diameter of the sphere corresponding to the commutativity domain transforms in the same way under the action of the maps, the transformation of the rest of the sphere does depend on the choice of extension.

The particular representation Eq. (29) corresponds to the expression used in the theorem considered in ref. 9. It is important however to stress, as also worked out in detail in the example below, that in the same work^9^, when discussing an example to support the result of the theorem, a different expression was in fact used to obtain a completely positive map starting from a zero discord correlated state and a given dynamics with respect to the one introduced in the proof. This point deserves clarification. Note that both Φ_1_(*t*) and Φ_2_(*t*) coincide when applied to states belonging to the compatibility domain, that is in this case diagonal in the basis {|*φ*_*i*_〉}_*i*_, while differ in their action on the rest of the states. This situation is schematically depicted in Fig. 1 with reference to the example treated below. In the figure the action of the two maps both on the commutative domain and on the whole Bloch sphere is depicted at the initial time and after finite time intervals. Note that the different action of reduced maps connected via the relation Eq. (27), as happens in this case, is actually amenable to experimental observation, and indeed the different evolution in time arising from reduced maps as in Eq. (27) has been exploited to experimentally detect initial correlations between system and environment^41^,^42^,^43^,^44^. The key point lies in the extension of the map from the initial domain to the whole set of states, which as we have shown can be performed in different ways. It is to be stressed that, besides acting differently on states outside the compatibility domain, the maps do generally not reduce to the identity for *t* = 0. This corresponds to the fact that the channels defined in Eq. (23) and Eq. (24), when composed with the partial trace with respect to the environmental degrees of freedom, act as the identity only on the compatibility domain, so that they do not define proper assignment maps^25^,^26^. This is a further signature of the fact that the extensions of the map do not allow for a clear physical interpretation.

### Correlated qubit states

The non-uniqueness of the proposed constructions can be better seen considering the following example. Let both 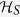 and 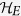 be isomorphic to *ℂ*^2^, and consider the correlated zero quantum discord state


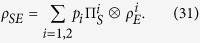


Denoting with {*σ*^0^, *σ*^1^, *σ*^2^, *σ*^3^} ≡ {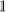, *σ*_*x*_, *σ*_*y*_, *σ*_*z*_} the basis of linear operators on *ℂ*^2^ made up of the identity and the Pauli matrices, we take 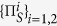 to be the projections on the eigenvectors of *σ*_*y*_ and assume 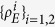 to be diagonal in the computational basis determined by the eigenvectors of *σ*_*z*_ corresponding to the eigenvalues +1 (|0〉) and −1 (|1〉), according to


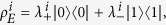


with 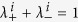. Let us further consider a unitary system environment interaction of the form


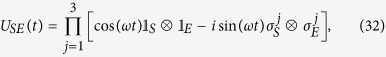


which allows for an analytic evaluation of the time evolution maps. The Choi matrices associated to these mappings can be easily obtained exploiting the relation Eq. (52). Neglecting time arguments for simplicity they can be compactly written


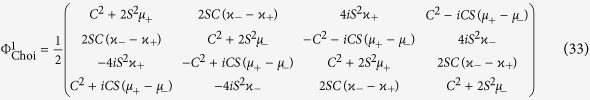


together with


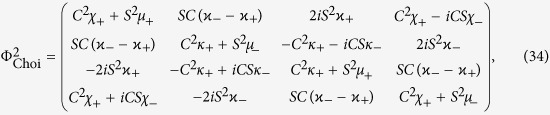


where we have denoted *C*(*t*) = cos(2*ωt*), *S*(*t*) = sin(2*ωt*) and the different constants are functions of the eigenvalues of the environmental statistical operators as detailed in the Methods Section, see Eq. (50). We now compare in detail these results with the analysis performed in ref. 9, where the problem of obtaining a completely positive mapping starting from a correlated state was addressed for zero discord states. In that paper the authors considered states of the form


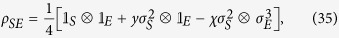


which having zero discord can be recast in the form Eq. (31) upon setting


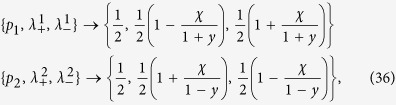


where the constraints 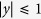 and 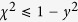 hold, and let it evolve according to Eq. (32). In particular by substituting the values Eq. (36) in the expression Eq. (50) one can obtain from Eq. (34) the corresponding expression for the Choi matrix, given in Eq. (54) of the Methods Section. It can be however noted that this matrix does not coincide with the result presented in Sec. 5 of ref. 9, despite the fact that the authors there advocate just one of the constructions that we used to come from a zero quantum discord state to the completely positive map, namely Eq. (29). The result can be understood as follows. The composition of any positive map, coming e.g. from a reduced dynamics as in Eq. (1), with a diagonalizing map as in Eq. (26) leads to a completely positive map, independently of the considered diagonalizing map. Indeed any diagonalizing map transforms the space of states in a commutative domain, on which positivity is equivalent to complete positivity, independently of the choice of orthogonal projections. As a result while in the proof of the theorem in ref. 9 a specific construction has been considered, in order to obtain a completely positive transformation, in the exemplification of the theorem a different diagonalizing map is considered.

### Reduced dynamics from discordant states

As simplest example of a completely positive map obtained starting from discordant states let us consider 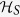 of dimension *n* and take the state


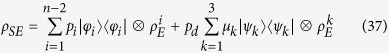


where introducing the two orthonormal states {|0〉, |1〉}, further orthogonal to {|*φ*_*i*_〉}_*i*=1, …, n−2_, we have {|*ψ*_1_〉 = |0〉, |*ψ*_2_〉 = |1〉, |*ψ*_3_〉 = |+〉}, with 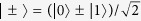. The state *W* can therefore be written


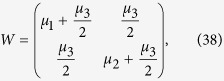


and one can consider its spectral decomposition


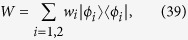


leading according to Eq. (16) to a set of six Kraus operators. For the simplest case of a uniform probability distribution, so that 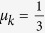 for all *k*, we have {|*ϕ*_1_〉 = |+〉, |*ϕ*_2_〉 = |−〉} together with {*w*_1_ = 2/3, *w*_2_ = 1/3}. This choice of parameters allows us to make direct contact with the example considered in ref. 12. We obtain in particular the set of Kraus operators 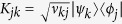



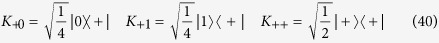



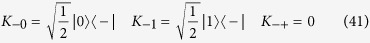


which leave *W* defined in Eq. (39) invariant according to


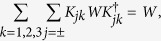


and leading according to the general theory to the positive operator-valued measure 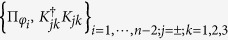. Note that this set of Kraus operators does not coincide with those exhibited in ref. 12. This fact can again be traced back to the non-uniqueness in the construction of the completely positive map. This is easily understood in our approach which rather than starting from a possible Kraus representation of the map proves its complete positivity based on its positivity on the physically relevant compatibility domain which is actually commutative. Indeed while the action of the map on the set of operators commuting with the marginal of Eq. (37) is uniquely defined, the extension to the whole space of statistical operators can be obtained in many ways, still preserving complete positivity. In particular it can be seen that the set {*K*_*jk*_} with 
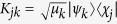
 still obeys Eq. (17) for any collection {*χ*_*j*_}_*j*_ of normalized but not necessarily orthogonal states such that 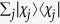 acts as the identity on the space spanned by {*ϕ*_*i*_}_*i*=1,2_.

## Discussion

The possibility to consider a reduced dynamical description for a given set of quantum degrees of freedom interacting with some external environment provides a very convenient way to account for the observed dynamics of such degrees of freedom. In this respect open quantum system theory has led to a satisfactory explanation of various physical phenomena and its general framework provides viable schemes to cope with the description of dissipation and decoherence effects in many different fields. However according to the general theory it is clear how to obtain a reduced dynamics only in the presence of an initially factorized system-environment state, a condition which cannot always be considered realistic in the presence of strong coupling. The extension of the formalism to include correlated initial states however appears to be non trivial and not always bears with itself the desired properties. In this article we have provided the construction of a dynamical map, for an arbitrary unitary interaction between system and environment, for a class of correlated states possibly including states with non zero discord. This set of states can be characterized as separable states with commuting marginals on the side of the system. This result encompasses previous work and puts it within a unified viewpoint, pinpointing the commutativity of the compatibility domain as the condition for the very introduction of a reduced map. It also shows that the definition of a reduced dynamical map in the case of correlated states is linked to the introduction of a set of Kraus operators building up a positive operator-valued measure arising from the transformation which leaves the reduced system state invariant. The key observation lies in the characterization of the set of reduced states compatible with given correlations. If this compatibility domain is made up of commuting states, exploiting the identification between positivity and complete positivity on such sets one can actually introduce well defined evolution maps. The latter can also be extended to the whole set of statistical operators, still retaining the property of complete positivity. However such extensions are generally highly non unique, as we have explicitly pointed out by means of example. This point, to the best of our knowledge, has yet not been put into the due evidence in the literature, and has allowed us to better clarify previous special results^9^,^12^, as explicitly discussed by means of example. Indeed while coinciding in their action on the compatibility domain, they generally transform in a different way states outside this domain. Moreover outside the compatibility domain they do not necessarily act as the identity at the initial time, thus describing a kind of initial slippage. The obtained picture, while elucidating a few basic points, and providing a constructive approach, further shows that extension of such maps beyond their natural domain, while preserving complete positivity is not necessarily of direct physical relevance.

It remains an open and relevant question whether the formalism can also be extended to states containing quantum correlations in the form of entanglement.

## Methods

### Construction of the mappings Φ_1_ and Φ_2_

We now consider how to explicitly obtain the completely positive maps Φ_1_(*t*) and Φ_2_(*t*) starting from the correlated state considered in Eq. (31), according to the dynamics described by Eq. (32). To this aim let us first rewrite Eq. (23) and Eq. (24) in such a way that the completely positive of the channels 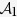 and 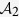 immediately appears


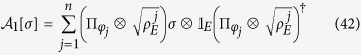



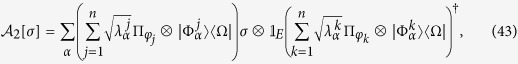


where |Ω〉 is an arbitrary normalized vector in 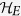. In order to identify the completely positive maps Eq. (28) and Eq. (29) we exploit a matrix representation of these maps^45^, given by


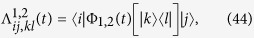


where the indexes take on the values 0 and 1. To actually evaluate the matrix elements we observe that the unitary evolution given by Eq. (32) can be written, up to an irrelevant phase factor, in the form


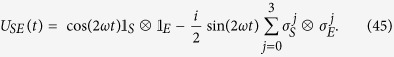


A straightforward but lengthy calculation then leads to the explicit expression for the matrices 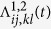, which act identically on states diagonal in the eigenbasis of *σ*_*y*_, while transforming in a different way system states diagonal in different bases. In particular they generally do not act as the identity map for *t* = 0.

We start considering the matrix associated to Φ_2_(*t*). We need to evaluate the operators


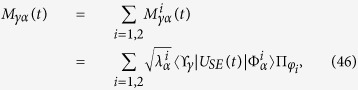


where 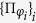 now denote the projections 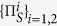 on the eigenvectors of *σ*_*y*_ and 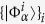 are actually independent from *i*, since both environmental statistical operators are diagonal in the computational basis. We obtain


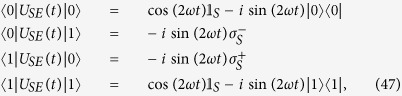


where we have denoted *C*(*t*) = cos(2*ωt*), *S*(*t*) = sin(2*ωt*) and defined the raising and lowering operators according to 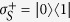 and 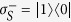, leading according to Eq. (30) to


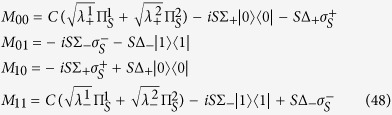


One can directly check the identity ∑_*γ*,*α*_*M*_*γα*_(*t*)^†^*M*_*γα*_(*t*) = 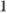
_*S*_, granting trace preservation of the map. Computing the action of the map on the computational basis by evaluating 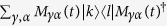 and taking the matrix elements one can find the matrix 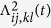



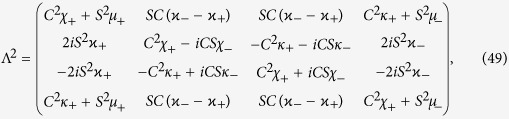


upon introducing the notation


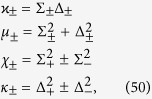


where


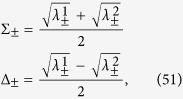


so that the constraints *μ*_−_ + *μ*_+_ = 1 and *κ*_+_ + *κ*_+_ = 1 are fulfilled.

### Choi matrices

Given this matrix representation complete positivity can be checked using the fact that the Choi matrices 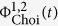 associated to the two maps are simply obtained by a suitable transposition of indexes


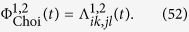


The expression of 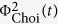 is given in Eq. (34), and its positivity can be directly checked.

The matrix representation of 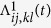 can be more simply obtained exploiting the result Eq. (27), and therefore evaluating the action of the Kraus operators *M*_*γα*_(*t*) on the elements 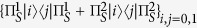, leading to the result


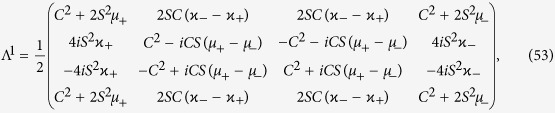


which further leads to the associated Choi matrix 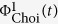 given in Eq. (33).

We note in particular that for the choice of system-environment state given by Eq. (36) upon substituting in Eq. (50) one recovers the following Choi matrix Eq. (34) associated to Φ_2_(*t*)


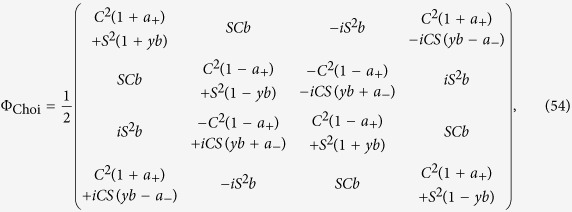


where to simplify notation we have set


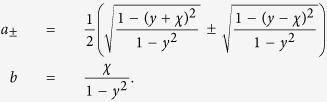


## Additional Information

**How to cite this article**: Vacchini, B. and Amato, G. Reduced dynamical maps in the presence of initial correlations. *Sci. Rep.* **6**, 37328; doi: 10.1038/srep37328 (2016).

**Publisher’s note:** Springer Nature remains neutral with regard to jurisdictional claims in published maps and institutional affiliations.
